# Spatial variability of soil fertility parameters in Kaski District, Nepal: A geostatistical approach

**DOI:** 10.1371/journal.pone.0360670

**Published:** 2026-11-05

**Authors:** Karishma Bhusal, Yojana Adhikari, Nirajan Acharya, Sadikshya Pokharel, Sunil Pandey, Rabindra Adhikari

**Affiliations:** 1 Soil and Fertilizer Testing Section, Province Agriculture Laboratory, Ministry of Agriculture, Land Management and Cooperatives, Pokhara, Kaski, Gandaki Province, Nepal; 2 Department of Soil Science, Agriculture and Forestry University, Rampur, Chitwan, Bagmati Province, Nepal; 3 Department of Agrigenomics, Christian-Albrechts-Universität zu Kiel, Kiel, Germany; 4 Institute of Forestry (IOF), Pokhara Campus, Tribhuwan University, Pokhara, Nepal; Ardakan University, IRAN, ISLAMIC REPUBLIC OF

## Abstract

Mapping of soils and depicting spatial variability across agro-ecological regions remains underexplored, especially in Nepal’s hilly areas. The current study aimed to assess spatial variability and map key soil properties such as pH, soil organic matter, total nitrogen, available phosphorus, and available potassium across the Kaski district, Nepal. A total of 263 composite, geo-referenced samples from 0–20 cm depth were collected using a proportional random sampling design across different land- use types. Fertility maps were generated using Inverse Distance Weighting (IDW) interpolation in ArcGIS 10.8. Descriptive statistics revealed significant spatial variability among soil parameters, with a coefficient of variation from 15.2% (low) for pH to 137.93% (high) for available phosphorus. Most soils were moderately to strongly acidic with a mean of 5.48 ± 0.004. Soil organic matter, total nitrogen, and available phosphorus showed medium fertility levels (SNI = 1.91, 2.19 & 1.95), whereas available potassium reflected a low soil nutrient index (SNI = 1.61), indicating potassium deficiency. Spatial autocorrelation analysis confirmed significant positive clustering for SOM and potassium, while nitrogen exhibited a random distribution pattern (I = 0.25, z = 1.46, p = 0.14). Trend analysis revealed directional gradients with saddle-shaped curves in pH, SOM, and total nitrogen, influenced by topography, elevation, and fertilizer management practices. This study provides a comprehensive characterization of soil fertility, highlighting fertility constraints and offering insights for precision agriculture and land-use planning aimed at sustainable soil management in the Kaski district of Nepal.

## Introduction

Soil is a key component of an ecosystem that influences many ecological processes through its physical, chemical, and biological roles. Conducting detailed studies and inventories of soil resources is essential for land use planning [1]. In Nepal, there is significant variation in climate, parent materials, soil properties, landscape, and physiography, which changes with altitude [1]. Several intrinsic factors, such as parent materials and climate, along with extrinsic factors like soil management practices, inherent fertility levels, and crop rotation, appear to affect soil properties across different areas [2]. The appropriate nutrient application rates should account for the crop’s nutrient requirements as well as soil fertility [3]. Fertile soil enhances soil nutrient cycling, improves structure by increasing organic matter accumulation, maintains balanced cation exchange, and lowers soil ESP to an acceptable range.

Soil fertility is a dynamic attribute influenced by both natural and human factors [4]. Soil fertility assessment provides knowledge on the available nutrients and the soil’s capacity to supply plant nutrients consistently. Understanding soil fertility status is a prerequisite for sustainable land use planning and designing crop cultivation strategies in the proposed area [5]. It is a critical determinant of soil productivity. Various soil fertility management programs, such as the use of organic manure, composting, green manure application, and soil testing, have been implemented by the Department of Agriculture in Nepal as part of an integrated plant nutrient management approach. Soil testing serves as a vital tool for assessing nutrient availability and directing sustainable fertility management [6]. Soil testing measures the soil’s fertility status and helps establish fertilizer recommendations for crops [7]. Agricultural land is currently degraded by soil acidity, nutrient unavailability, and low levels of organic matter. GIS and GPS technologies are now used to describe the spatial variability of soil. Employing GPS in soil sample collection is important for creating accurate soil fertility maps [8]. It is a promising tool for location-specific monitoring of soil nutrient status [9].

A Geographic Information System (GIS) is a tool that transforms voluminous data on natural resources, making it easier to interpret, which would otherwise be difficult to handle manually. It simplifies diverse and attributed data into a form that facilitates understanding and eases data handling [10]. Tools such as GIS, GPS, remote sensing, and various statistical techniques expand the scope of data collection, analysis, and prediction of soil spatial information. They can be used to store, manipulate, and analyze large volumes of spatial data [11]. Soil maps have been used for land valuation, crop selection, and cultivation planning [12]. They facilitate easier decision-making means for researchers, farmers, students, and land-use planners for sustainability design and land-use management if integrated with the Decision Support System [13]. Digital soil mapping provides the statistical models of the interrelations between environmental covariates and soil attributes [14]. Soil fertility maps are significant for addressing resource management issues, such as soil erosion and degradation, as well as for urban planning. Testing soil parameters over large areas is costly, inefficient, and yields non-representative results. In the past, soil mapping relied on field observations and laboratory analysis of samples, which limited scalability, precision, accuracy, and cost optimization. In recent years, several notable advancements have been made in digital soil mapping. GIS–based soil fertility maps provide easy and effective nutrient management strategies across large regions [15]. GIS enables the integration of field data and laboratory analysis into a single geodatabase that contains both attributes and spatial information [16]. GIS and GPS technologies are widely adopted for site-specific nutrient management and proper land management. They explain the distribution of plant nutrients and the patterns of nutrient availability and depletion within the mapped area [1]. Interpolation techniques, IDW (Inverse Distance Weighting), and Kriging are widely adopted to develop high-resolution maps of distant, varied places and to estimate their soil properties. IDW was selected owing to the sparse and random distribution of sampling points in the study area, as described in the Statistical Analysis subsection of Materials and Methods.

Detailed soil nutrient maps are important for identifying areas with fertility constraints and helping farmers implement agricultural management measures to improve crop productivity. Few studies have mapped soil physical and chemical properties at the district level in Nepal. Studies with detailed spatial variability analysis are limited, as the landscape in the hilly regions has high variability in the shortest distance [17]. Reliable and updated soil fertility maps of many regions of Nepal are not available for study [14]. It is important to use accurate geostatistical methods to study the spatial structures. This gap can be filled by field sampling and presenting soil fertility parameters in the form of digital maps for easy farmers’ access and high value to them [18].

Despite the importance of soil fertility mapping, detailed spatial variability studies at the district level in Nepal, particularly in hilly regions like Kaski, remain limited. The highly heterogeneous landscape demands fine-scale soil fertility assessment for effective nutrient management. Therefore, this study aimed to assess and map the spatial variability of key soil fertility parameters using geostatistical techniques and GIS-based digital soil mapping approaches in Kaski district, Nepal.

## Materials and methods

### Study area

Kaski district is located in the mid-hill ecological zone of central-western Nepal. It is situated in Nepal’s hilly region, within Gandaki Province, and spans a total area of 2,017 km^2^ [19]. The study area (Fig 1) includes all local administrative units within Kaski District, covering various agro-ecological zones. The district exhibits marked soil heterogeneity associated with its steep gradient, with elevation ranging from the Terai lowlands (450 m) to the mountainous Annapurna region (8091 m) above sea level.

**Fig 1:**
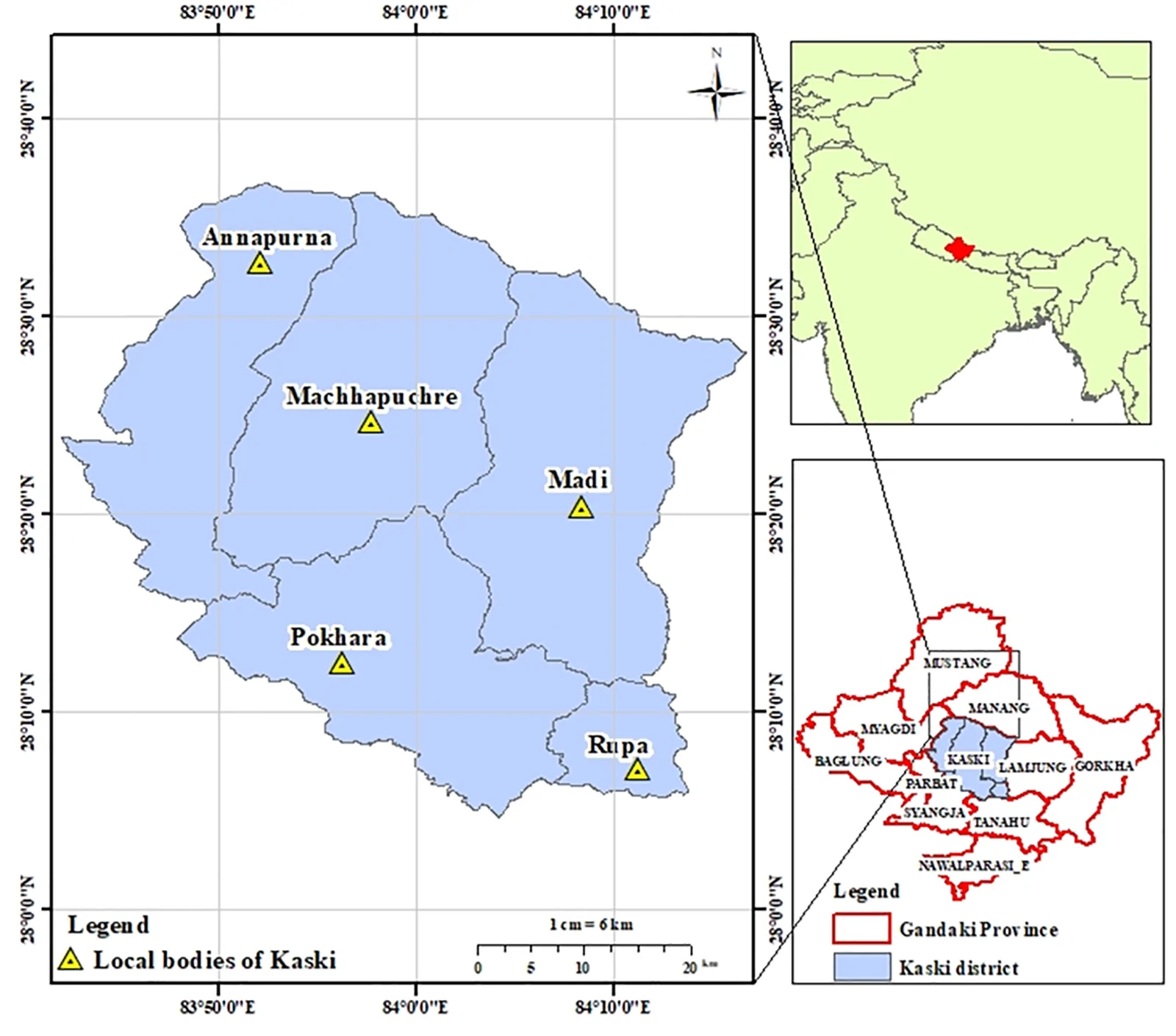
Location map of the study area, Kaski district, Nepal. The world boundary data were sourced from Natural Earth (public domain) and national, provincial and district administrative boundary data were sourced from the National Geoportal of Nepal. The map was generated and prepared by the authors using Arc GIS 10.8.

Soils like Entisols, Inceptisols, Alfisols and Mollisols are prominent under FAO World Reference Base for Soil Resources, cross-referenced with USDA Taxonomy, which corresponds to Cambisols and Luvisols in the middle mountain zone of Pokhara and lower regions of Machhapuchhre, Rupa, and Madi. The higher mountain zones of upper Annapurna and Machhapuchre are dominated by Entisols and Inceptisols under USDA Taxonomy, classified as Dystric Regosols and Umbrisols under WRB characterization, symbolizing less developed soils in high-altitude slopes. Likewise, Alluvial soils along the Seti River and Pokhara valley are classified as Calcaric and Gleyic Fluvisols, corresponding to Eutric Cambisols and Gleysols [20,21]. Agricultural land makes up about 24% of the total area, while forest land accounts for approximately 46%. The district receives exceptional rainfall, ranging from 3000 mm to over 4000 mm depending on the locations [22], and has a temperature range of 13–24 °C.

### Sample collection and laboratory analysis

Soil sampling was conducted across different land-use types within the study area to evaluate fertility parameters related to crop production. The sampling was conducted by the Province Agriculture Laboratory, as a part of its official soil survey program, in coordination with the Agriculture Sections of the respective local bodies. No special permits were required for the access and collection of soil samples since both the institutions fall under government authorities. Each local administrative unit in the district was classified based on area, slope, and elevation, with a proportional random sampling method applied within each class. A total of 263 composite samples were collected from a depth of 0–20 cm using a soil auger at selected locations, with the geographic coordinates of each sampling point recorded using a Garmin GPS device (Fig 2). The top 20 cm soil depth is an active root zone for most annual crops like cereals, pulses and vegetables, and plays a primary role in deciding soil sampling depth as it is relevant to crop nutrient uptake [23]. The sampling design considered both agricultural land and other land-use types for comprehensive spatial analysis. The samples were collected from the field, bagged, sealed, and labelled in zipped plastic bags. Additionally, sample preparation for analysis was performed in the laboratory, for which the soil particles were dried at room temperature, ground, and sieved through a 0.2 mm mesh for organic matter and through < 2 mm for other parameters. The samples were then analyzed at the Province Agriculture Laboratory (Soil and Fertilizer Testing Section), Gandaki Province, Pokhara, Nepal, for routine soil fertility parameters, including pH, total nitrogen (TN), phosphorus (P_2_O_5_) and potassium (K_2_O), and soil organic matter (SOM), following standard laboratory methods and procedures (Table 1). The nutrient levels are rated in order (high, medium, low) for each parameter, with the range of values as given by the National Soil Science Research Center, Khumaltar, Nepal [24] (Table 2). Fig 3 illustrates the overall design and workflow of the adopted methodology.

**Table 1:** Laboratory methods used for testing of different soil chemical parameters.

S.N.	Parameters	Unit	Methods
**1**	**Soil pH**	–	1:2.5 Potentiometric method [25]
**2**	**Soil organic matter (SOM)**	%	Walkley-Black method [26]
**3**	**Total Nitrogen (TN)**	%	Kjeldahl method [27]
**4**	**Available phosphorus (P**_**2**_**O**_**5**_)	kg/ha	Modified Olsen’s Bicarbonate Method [28]
**5**	**Available potassium (K**_**2**_ **O)**	kg/ha	Ammonium acetate method [25]

**Note**: kg/ha is kilogram per hectare.

**Table 2:** Rating of different soil parameters given by NSSRC as reported by [24] for the hilly region of Nepal.

Parameter	Unit	Range	Rating
pH		<4.5	Extremely acidic
		4.5-5.5	Very strongly to strongly acidic
		5.5-6.5	Moderately to slightly acidic
		6.5-7.5	Neutral
OM	%	>7.5	Alkaline
		<2.5	Low
		2.5-5	Medium
		>5	High
TN	%	<0.1	Low
		0.1-0.2	Medium
		>0.2	High
P_2_O_5_	kg/ha	<30	Low
		30-55	Medium
		>55	High
K_2_O	kg/ha	<110	Low
		110-280	Medium
		>280	High

**Fig 2:**
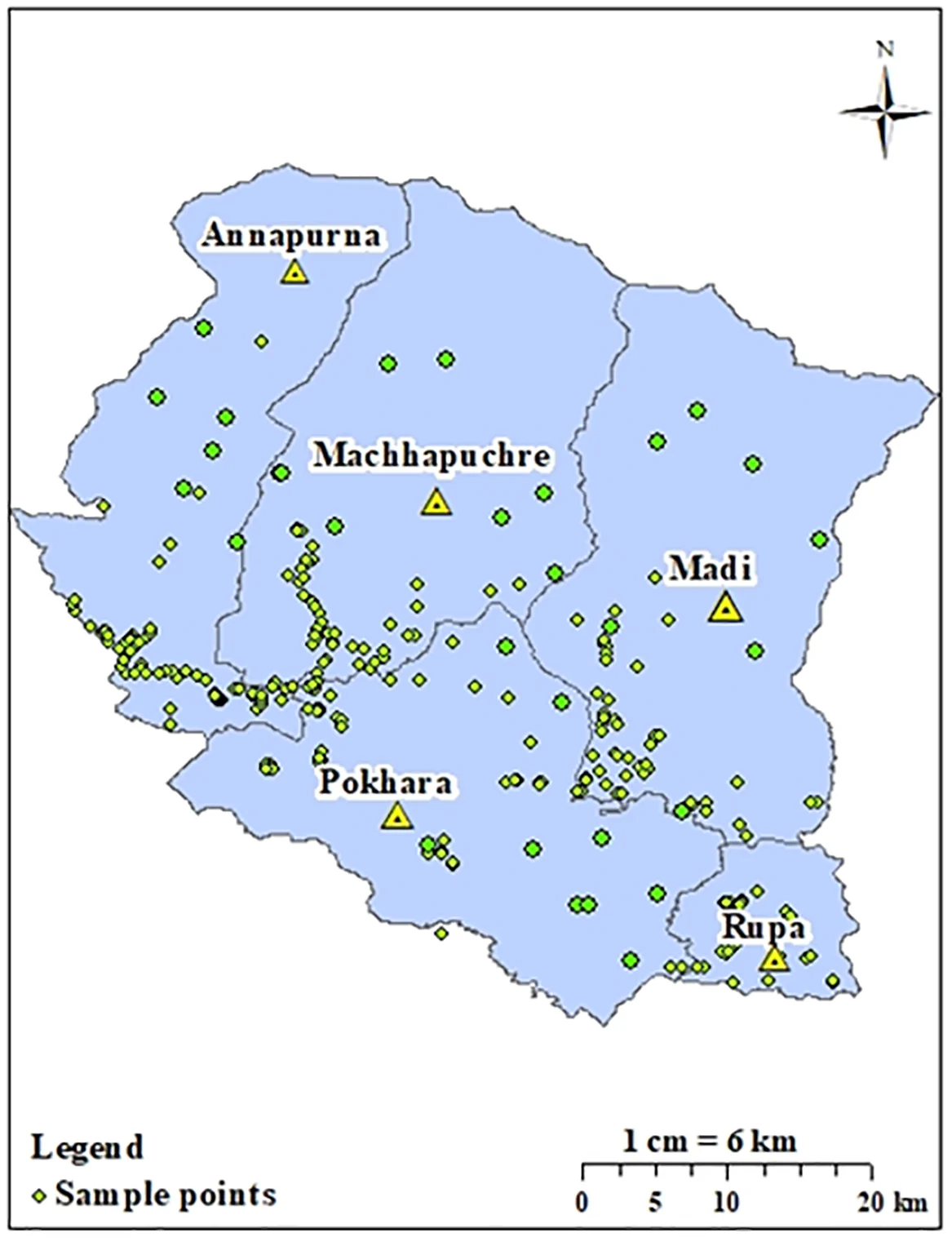
Distribution of georeferenced soil sampling points in the study area. The district administrative boundary data were obtained from the National Geoportal of Nepal. The map was generated and prepared by the authors using Arc GIS 10.8.

**Fig 3:**
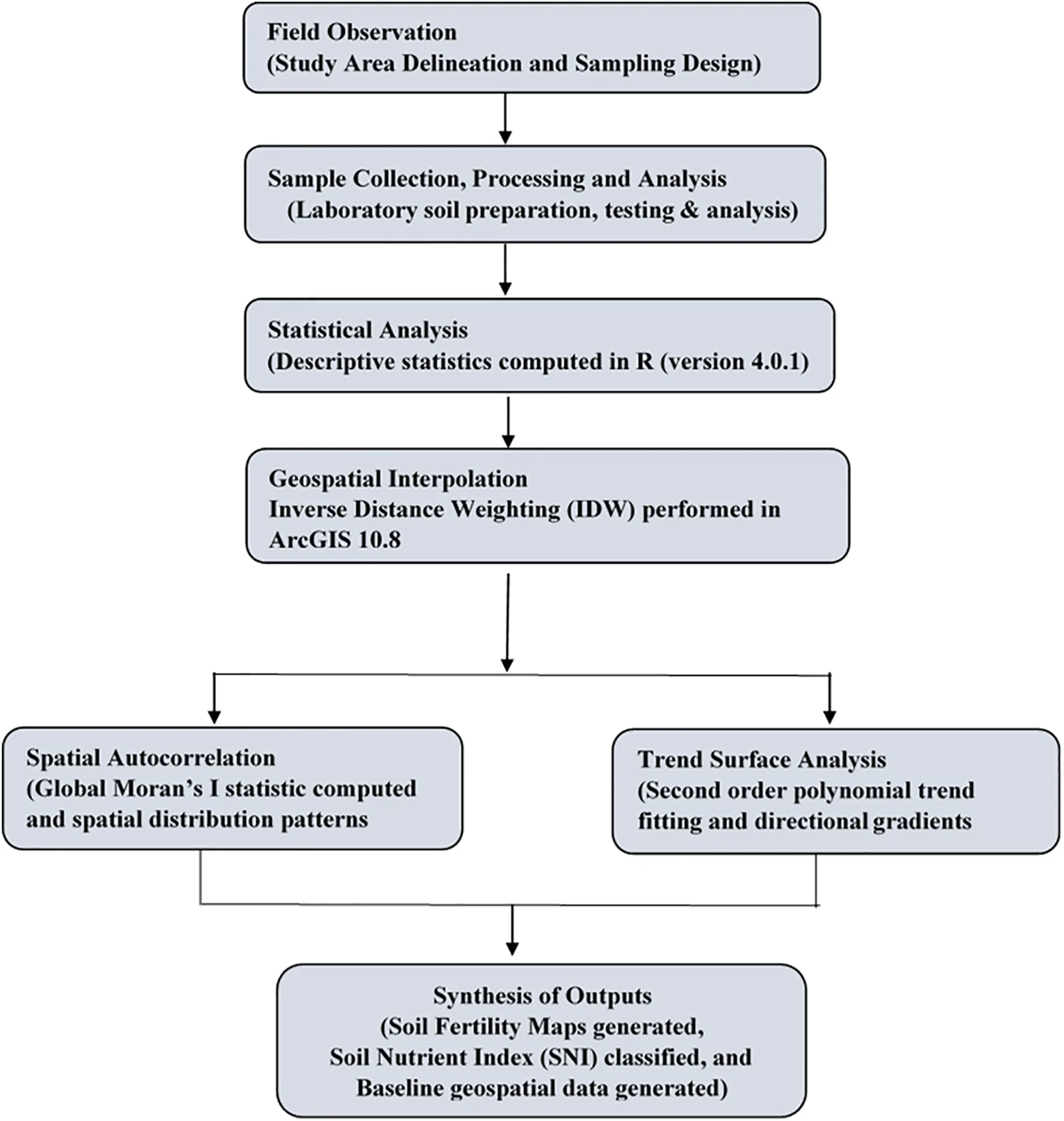
Schematic representation of the experimental design and workflow adopted.

### Statistical analysis

Soil chemical properties for each parameter (pH, SOM, TN, P2O5, K2O, SNI) were analyzed using descriptive statistics, including mean, median, maximum, minimum, standard deviation, standard error of the mean, and coefficient of variation, in RStudio using R version 4.0.1. Additionally, the coefficient of variation was categorized into three levels: < 10, 10–100, and >100, corresponding to low, moderate, and high, respectively [17].

Skewness and kurtosis were calculated for soil parameters to assess the normality of the data distribution, and a log transformation was applied to non-normal data to restore normality. Spatial datasets were referenced to GCS Nepal Nagarkot based on the D Nepal Nagarkot datum. The layers were then projected to the World Geodetic System (WGS) 1984 Universal Transverse Mercator Zone 45N before spatial analysis, as it minimizes distortion for the study locations. Soil fertility maps were created in ArcMap 10.8 using ArcGIS spatial analysis tools. The interpolation method used between sampled locations was Inverse Distance Weighted (IDW). The sampling points in the area were relatively sparse and unevenly distributed, so IDW was chosen over other methods. IDW is well suited for interpolating randomly distributed and sparse data in vegetation studies while Kriging is for well-distributed dense data [29,30]. Additionally, IDW is easy to use and has low computational load [31] and doesn’t require the sample density to reliably estimate a semivariogram model [32] and is also suitable under conditions of high spatial autocorrelation compared to Kriging [33].

### Spatial autocorrelation

The Global Moran’s I statistic measures spatial autocorrelation in data, accounting for the locations of features and their corresponding attribute values. It evaluates the spatial distribution pattern, whether it is clustered, dispersed, or random, and reflects the influence among neighboring sample points. Soil sampling locations with their chemical properties were assigned to the spatial database and Global Moran’s I analysis was done using the spatial autocorrelation tool in the ArcMap, Moran’s I index values, z-values, and p-values were generated to identify the degree and statistical significance of spatial autocorrelation among the studied soil parameters [34].

#### Soil nutrient index.

The soil nutrient index was estimated using the formula proposed by [35].

$\text{Nutrient\textasciitilde{}Index\textasciitilde{}(NI)}=\text{1}\times N_{L}+\text{2}\times N_{M}+\text{3}\times N_{H}\;/{\;N}_{T}$

Where ${\text{N}}_{\text{L}}{,\text{ N}}_{\text{M},\;}{\text{ and N}}_{\text{H}}$ represent the number of samples categorized under low, medium and high nutrient status, respectively and${\text{ N}}_{\text{T}}$ represents the number of total analysed samples for a given area of study. The interpretation of the values was in line with that of [35] as shown in Table 3.

**Table 3:** Soil nutrient index (SNI) ranges.

Soil Nutrient Index (NI) ranges	Remarks
Below 1.67	Low
1.67-2.33	Medium
Above 2.33	High

### Trend analysis

Trend analysis in ArcMap is a data analysis technique that shows a three-dimensional orientation of the spatial data to identify directional trend by fitting the polynomials through the scatter plots [36]. It projects the data on two perpendicular planes, including the X-Z and Y-Z planes. The green and blue lines depict the trends along the X-Z (E-W) and Y-Z (N-S) planes, respectively. A straight line indicates the theoretical (normal) distribution, and a U-shaped curve shows the directional trend existing in the sampled data. The data must be subjected to analysis of its second-order polynomial to generate an accurate interpolated map [37].

## Results and discussion

The soil fertility of the studied area was assessed based on soil chemical properties, including pH, organic matter, and primary nutrients such as nitrogen, phosphorus, and potassium. The statistical summary of all these chemical properties is presented in Table 4 and discussed under the following headings. Dispersed CV values indicate differences in soil nutrient properties across the study area.

**Table 4:** Descriptive statistics of soil chemical properties of Kaski district, Nepal.

Parameter	Unit	Min.	Max.	Mean	SD	SEM	CV(%)	Skewness	Kurtosis	Median
**pH**	–	3.7	7.4	5.48	0.83	0.004	15.2	0.08	−0.89	5.45
**SOM**	%	0.6	6.58	3.5	1.49	0.09	42.55	0.32	−0.86	3.27
*SOM	%	−0.22	0.82	0.5	0.21	0.01	41.87	−0.68	0.39	0.51
**TN**	%	0.03	0.33	0.17	0.07	0.004	42.94	0.29	−0.84	0.16
**P**_**2**_ **O**_**5**_	kgha^-1^	0.59	934.61	84.39	116.4	7.18	137.93	2.95	12.77	43.74
*P_**2**_ **O**_**5**_	kgha^-1^	−0.23	2.97	1.58	0.59	0.04	37.62	−0.17	−0.44	1.64
**K**_**2**_ **O**	kgha^-1^	2.4	1406.4	169.93	200.8	12.39	118.2	2.35	7.36	88.8
*K_**2**_ **O**	kgha^-1^	0.38	3.15	1.96	0.51	0.03	26.18	−0.24	−0.18	1.95
										

### Descriptive statistics

The descriptive statistics of soil parameters are presented in Table 4. Phosphorus and Potassium demonstrated high spatial variability (137.9 and 118.2%) among all, while the pH of soil samples showed low variability (15.2%). In addition, available phosphorus and potassium were highly skewed (2.95 & 2.35, respectively) and had high kurtosis values (12.77& 7.36), indicating non-normal distribution with the existence of outliers. Soil organic matter (SOM) exhibited slight positive skewness with a relatively flat distribution (kurtosis = −0.86). Log transformation was applied to them as *P_2_O_5_ and *K_2_O, to retrieve their data in normal distribution (skewness values of −0.17& −0.24 and kurtosis values of −0.44 & −0.18 respectively) before projecting them to statistical analysis and interpolation.

### Spatial variability of soil chemical properties

#### Soil pH.

Soil pH is the primary soil characteristic that influences the different physicochemical and biological soil components and crop productivity [7]. It plays a vital role as a soil regulator and controls the function of other soil-forming variables [38]. The soil pH varied from 3.7 to 7.4, with a mean value of 5.48 ± 0.004 (Table 4). Soil mapping of pH in Kaski district represented that most soils are in the moderate (5.5–6.5) to strongly acidic class (4.5–5.5) and very few are in the neutral range (Fig 4a). No sample from the Madi rural municipality showed the neutral soil pH (6.5–7.7) value. Similarly, the soils of Pokhara Metropolitan city were found slightly acidic to neutral in class (Fig 4a), with very few under the highly acidic class, which concurs with the results of [39]. The parameter pH showed minimum variability (15.2%) among the soil samples (Table 4).

**Fig 4:**
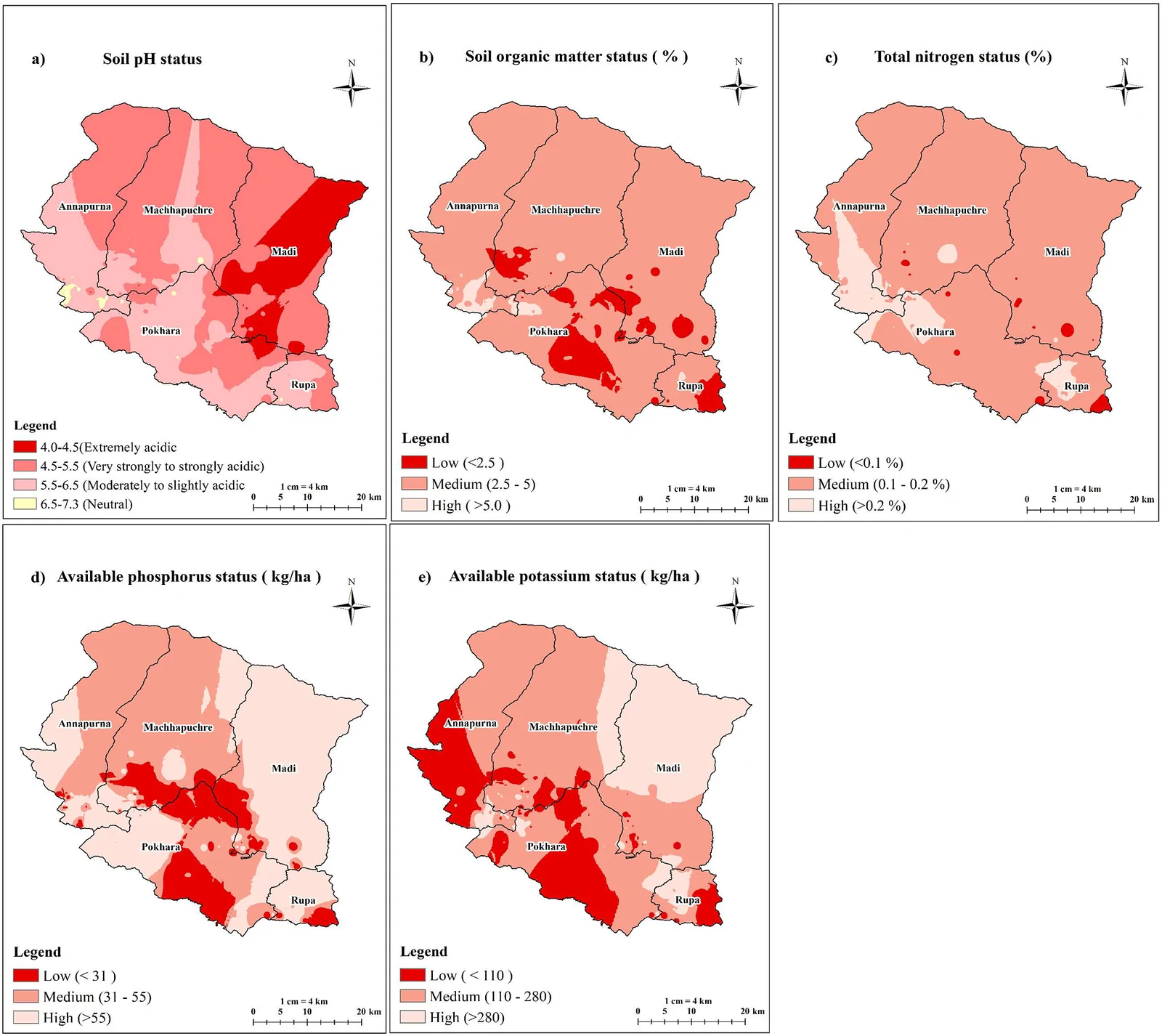
Spatial distribution map of soil parameters a) soil pH, b) Organic matter, c) Nitrogen, d) Phosphorus, e) Potassium. The district administrative boundary data were obtained from the National Geoportal of Nepal. The map was generated and prepared by the authors using Arc GIS 10.8.

#### Soil organic matter.

Soil condition can be improved by maintaining organic matter levels. It is a key factor regulating and improving the soil’s physical, chemical, and biological properties and forms a larger part of soil [40]. The soil organic matter content ranged from low to very high (0.6 to 6.58%), with an average of 3.5% (Table 4). Organic matter showed moderate spatial variability (42.55%) among the soil samples. The studied soil samples showed medium levels of organic matter being more common across local areas of the district (Fig 4b). The soils of the Madi rural municipality are only in the medium- high range of organic matter status (Fig 4b). The cooler temperatures at higher altitudes slows down microbial decomposition of organic substances, which explains the optimal organic matter levels shown on the map [41]. However, the low status may be the outcome of poor incorporation of organic manures, ignorance to resource conservation technologies including leaving of crop residues in the field, soil turning and mechanical tilling, sustainability practices and high mineralization of organic matter [5].

#### Total nitrogen.

Nitrogen is the major limiting nutrient required for crops in every aspect of growth. The total nitrogen content varied from 0.03% to 0.33%, i.e., low to very high, with an average of 0.17 ± 0.004 (Table 4). Nitrogen status was recorded with a variability of 42.94% (Table 4). The nitrogen properties were reported to be similar [42]. Among all the local bodies of Kaski district, the soils of Madi rural municipality recorded a low to medium range of nitrogen, with very few areas under high status. Likewise, the soils of Annapurna rural municipality showed medium to high nitrogen status and no indication of a lower range (Fig 4c). The nitrogen level, in the majority of the area, especially in high altitudes, as observed in the fertility map, is in the medium range (Fig 4c). Total nitrogen rises with altitude due to the lower rate of mineralization, low temperature and more accumulation of organic matter [43]. Differences in soil management practices, fertilizer application methods, and doses result in variability in total N content [39]. The lower cultivable areas record a major deficiency in the total nitrogen due to its susceptibility to loss through volatilization, runoff and leaching [44].

#### Available phosphorus.

After Nitrogen, phosphorus is the most limiting nutrient [45]. Phosphorus availability in soil is highly affected by soil pH, with a range between 6 and 7.5 being observed as the ideal one for phosphorus, but pH below 5.5 and above 7.5 causes fixation of soil phosphorus with aluminum, iron and calcium, lowering its availability (Ma et al., 2019). The available phosphorus in soil also varied from 0.59 to 934.61 kg ha^−1^, corresponding to a low to high class with a mean value of 84.39 ± 7.18 kg ha^−1^ (Table 4). It showed a high level of spatial variation (137.93%) across the study area [39]. also reported high variability in soil available phosphorus content across local bodies in Kaski. The distribution of soil available phosphorus ranged from low to high, with the minimum area of lower phosphorus level in Annapurna Rural Municipality and ea of higher phosphorus level in Madi Rural Municipality compared to other local bodies of Kaski district (Fig 4d).

#### Available potassium.

Potassium is the third major nutrient required primarily for a plant’s growth & its biochemical and physiological processes [46,47]. The concentration of available potassium ranged from 2.4 to 1406.4 kgha^−1^, falling within the low to very high class, with a mean of 169.93 ± 12.39 kgha^−1^ (Table 4). Overall, the low, medium and high status of potassium distribution is observed with a variability of 118.2 % indicates a high spatial dispersion in soil potassium levels across all areas of study (Fig 4e). The fertility map showed critically low potassium levels in high area coverage of Pokhara metropolitan city and Annapurna Rural Municipality (Fig 4e). The results align with [39], in which they reported the low potassium in soils of the Pokhara metropolitan of Kaski district. Soil acidity affects the availability of the potassium nutrient [48]. Similarly, the presence of organic matter and phosphorus also increases the potassium availability in the soil [49].

### Soil nutrient index (SNI)

The soil nutrient indices for different soil parameters (OM, N, P, and K) were observed as 1.91, 2.19, 1.95, and 1.61, respectively (Table 5). It showed that the potassium content status in soil samples was low compared to other nutrients, which were recorded as medium.

**Table 5:** Nutrient Index values for soil samples in Kaski district, Nepal.

Parameter	Total sample (T)	SNI	Remarks
**SOM**	263	1.91	Medium
**TN**	263	2.19	Medium
**P**_**2**_ **O**_**5**_	263	1.95	Medium
**K**_**2**_ **O**	263	1.61	Low

### Spatial autocorrelation

Global Moran’s I analysis presented in Table 6 confirmed significant positive spatial autocorrelation for SOM (I = 0.34, p < 0.01 & z-score = 2.44) and K_2_O (I = 0.37, p < 0.05, z-score = 2.75), indicating clustered distribution patterns. The spatial distribution of the potassium and organic matter is non-random, following a systematic, structured pattern. However, N and P_2_O_5_ didn’t show significant spatial dependence and weak clustering (p > 0.05) seemed more pronounced. In addition, the pH value showed a borderline p-value (0.05) and a z-score (1.94), demonstrating marginally significant spatial autocorrelation (Table 6). Nitrogen revealed the weakest and non-significant spatial autocorrelation (I = 0.25, p = 0.14 and z-score = 1.46) as shown in Table 6, where the variability may be associated with the management practices on site. The ordinary kriging method was used initially; however, the limited sampling density generated empty patches and unstable prediction surfaces. Hence, the IDW method of interpolation was applied to generate continuous spatial distribution maps.

**Table 6:** Spatial autocorrelation of soil properties.

Statistic	pH	SOM	N	P_2_O_5_	K_2_O
**Moran’s I**	0.27	0.34	0.25	0.24	0.37
**Variance**	0.019	0.019	0.029	0.018	0.018
**z-value**	1.94	2.44	1.46	1.79	2.75
**p- value**	0.05	0.01	0.14	0.07	0.005

### Trend analysis

Trend analysis was performed in ArcGIS 10.8 using the Geostatistical Analyst tool. Fig 5 illustrates the spatial distribution of soil sampling locations in the X and Y planes and soil chemical properties in the Z plane. As observed in Fig 4, a U-shaped curve in the trend plot shows a second-order polynomial that describes the systematic spatial variation across the study area.

**Fig 5:**
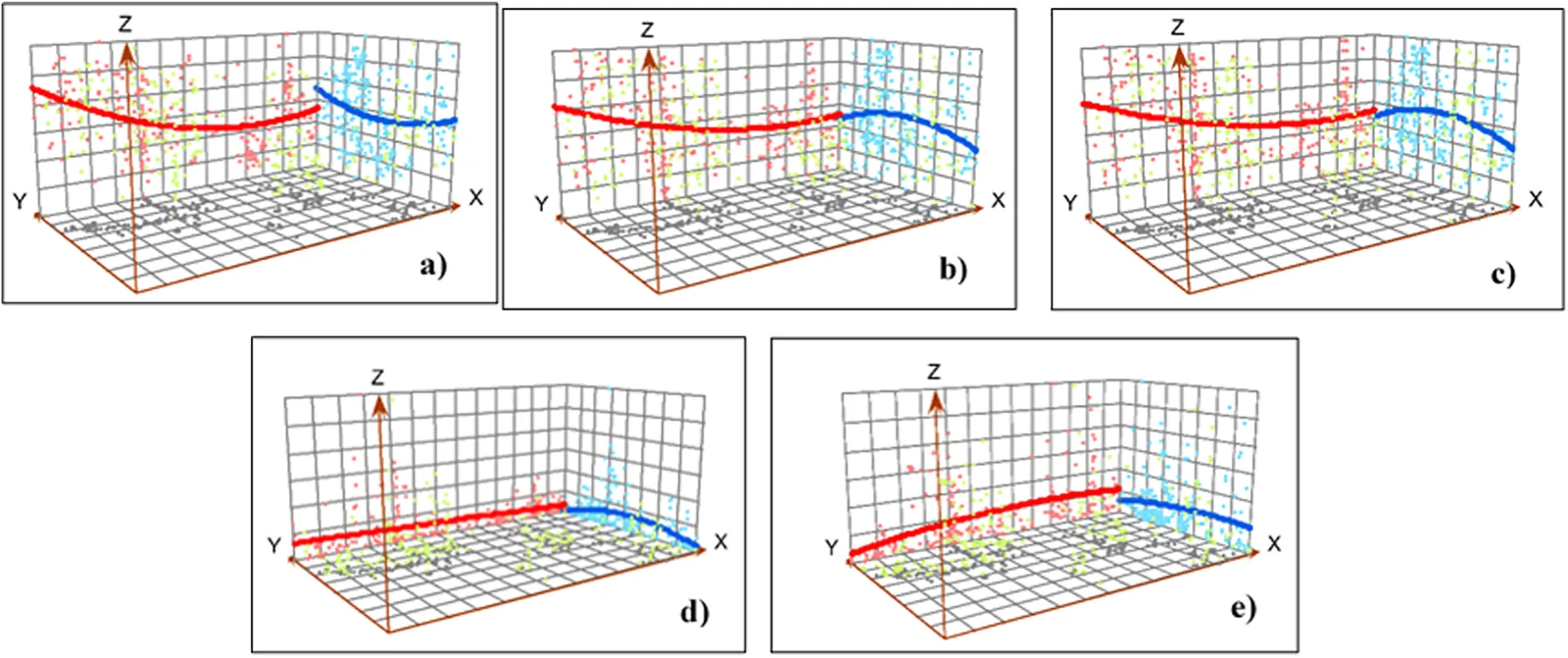
Trend analysis of the sampled data a) soil pH, b) Organic matter c) Nitrogen, d) Phosphorus, e) Potassium.

The results in Fig 5 revealed that among all the soil parameters, soil pH, organic matter, and nitrogen exhibited a pronounced directional trend effect. The parameters, including organic matter and nitrogen, depicted a similar trend of lower values in the middle along the N-S plane with increasing values along the edges and higher values towards the center along the E-W plane, with lower values along the edges. This saddle-shaped distribution reflects the variations in the factors such as vegetation cover, land use patterns, landscape and topography across the study area. In contrast, phosphorus and potassium depicted relatively uniform distribution with no significant directional trend (Fig 5).

## Conclusion

A clear understanding of the spatial distribution of soil properties across the landscape is essential for identifying nutrient-deficient areas, assessing nutrient availability and status, and guiding land-use decisions. Integrating GIS-based analysis with laboratory data proves to be an effective method for soil fertility assessment. The study evaluated the spatial variability of soil parameters and the region-specific challenges of soil acidity and nutrient imbalance in different local administrative units of Kaski district using GIS-based interpolation techniques in ArcMap. The integrated geostatistical mapping framework adopted in the study, combining IDW interpolation, spatial autocorrelation and trend analysis, guides a transferable methodology for categorizing soil properties across hilly and mountain regions having heterogeneous landscapes worldwide, where irregular sampling often constrains the use of conventional approaches like Kriging. The fertility maps also provide explicit evidence to provincial and municipal agriculture offices for planning targeted subsidy programs prioritizing the nutrient deficit zones. The findings also hold value for fertilizer industries and suppliers operating in the region in ensuring the availability of fertilizer blends tailored to the documented deficient nutrients. The saddle-shaped directional trends observed for pH, organic matter, and nitrogen levels demonstrated that topographic gradients, land cover and varied land-use across Kaski’s local bodies contribute to spatial heterogeneity in these parameters, rather than a uniform regional influence. Mapping showed low fertility in steeper, higher terrains of Kaski compared to lower elevation areas. There was high variability among soil samples for phosphorus and potassium, moderate variability for organic matter and nitrogen, and low variability for soil pH. The limited areas with neutral pH suggests the need for liming and consideration of alkaline supplements to stabilize pH levels. Additionally, the low nutrient index of soil potassium in the majority of samples indicates the necessity for potassium enrichment through proper fertilizer management and soil practices. Furthermore, the findings from the fertility maps can deliver baseline information for site-specific nutrient management, and help extension workers to make decisions on recommendations and optimize fertilizer inputs.

The study has some limitations, a limited number of samples, soil sampling in a single season and from the surface layer (20 cm depth), which accounts only for the surface soil horizons and less considers the temporal variability. Therefore, the study scope may not cover the seasonal fertility fluctuations and every aspect of soil health. The deployed IDW method may bring certain errors in the fertility map. Further studies should incorporate the higher sampling density, variable sampling locations, deeper soil profiles and also the inclusion of micronutrients to validate the method of mapping and findings. The extended evaluation would help to establish the reliability and comprehensiveness of the spatial soil assessment in the region.

## Supporting information

S1 Datafiles Compressed archive containing supplementary data files supporting this study. (RAR)
